# Test site predicts HIV care linkage and antiretroviral therapy initiation: a prospective 3.5 year cohort study of HIV-positive testers in northern Tanzania

**DOI:** 10.1186/s12879-016-1804-8

**Published:** 2016-09-20

**Authors:** Elizabeth A. Reddy, Chris Bernard Agala, Venance P. Maro, Jan Ostermann, Brian W. Pence, Dafrosa K. Itemba, Donna Safley, Jia Yao, Nathan M. Thielman, Kathryn Whetten

**Affiliations:** 1 Division of Infectious Disease, Upstate Medical University, Syracuse, NY USA; 2 Department of Health Policy and Management, University of North Carolina, Chapel Hill, NC USA; 3 Department of Medicine, Kilimanjaro Christian Medical Centre, Moshi, Tanzania; 4 Department of Health Services Policy & Management, Arnold School of Public Health, University of South Carolina, Columbia, SC USA; 5 Department of Epidemiology, University of North Carolina, Chapel Hill, NC USA; 6 Duke University Center for Health Policy and Inequalities Research, Durham, NC USA; 7 Tanzania Women Research Foundation, Moshi, Tanzania; 8 Duke Center for Health Policy and Inequalities Research, Duke University, Durham, NC USA; 9 Duke Global Health Institute and Duke Division of Infectious Diseases, Duke University, Durham, NC USA

**Keywords:** HIV testing, HIV linkage to care, HIV care continuum, Health services accessibility, Africa, Mental health, Resource limited settings, Antiretroviral therapy

## Abstract

**Background:**

Linkage to HIV care is crucial to the success of antiretroviral therapy (ART) programs worldwide, loss to follow up at all stages of the care continuum is frequent, and long-term prospective studies of care linkage are currently lacking.

**Methods:**

Consecutive clients who tested HIV-positive were enrolled from four HIV testing centers (1 health facility and 3 community-based centers) in the Kilimanjaro region of Tanzania as part of the larger Coping with HIV/AIDS in Tanzania (CHAT) prospective observational study. Biannual interviews were conducted over 3.5 years, assessing care linkage, retention, and mental health. Bivariable and multivariate logistic regression analyses were conducted to determine associations with early death (prior to the second follow up interview) and delayed (>6 months post-test) or failed care linkage.

**Results:**

A total of 263 participants were enrolled between November, 2008 and August, 2009 and 240 participants not already linked to care were retained in the final dataset. By 6 months after enrollment, 169 (70.4 %) of 240 participants had presented to an HIV care and treatment facility; 41 (17.1 %) delayed more than 6 months, 15 (6.3 %) died, and 15 (6.3 %) were lost to follow up. Twenty-six patients died before their second follow up visit and were analyzed in the early death group (10.8 %). Just 15 (9.6 %) of those linked to care had started ART within 6 months, but 123 (89.1 %) of patients documented to be ART eligible by local guidelines had started ART by the end of 3.5 years. On multivariate analysis, male gender (OR 1.72; 95 % CI 1.08, 2.75), testing due to illness (OR 1.63; 95 % CI 1.01, 2.63), and higher mean depression scale scores (4 % increased risk per increase in depression score; 95 % CI 1 %, 8 %) were associated with early death. Testing at a community versus a hospital-based site (OR 2.89; 95 % CI 1.79, 4.66) was strongly associated with delaying or never entering care.

**Conclusions:**

Nearly 30 % of the cohort did not have timely care linkage, ART initiation was frequently delayed, and testing at a hospital outpatient department versus community-based testing centers was strongly associated with successful care linkage.

## Background

A growing body of evidence has now demonstrated both the therapeutic and preventative benefits of early antiretroviral therapy (ART), increasing the global urgency to identify HIV-infected individuals and link them to care [1–4]. However, programmatic, psychosocial, sociodemographic, and illness-related barriers contribute to substantial attrition across the care continuum [5–7]. Poor care linkage appears to be particularly pronounced in sub-Saharan Africa, the world region most heavily affected by the global HIV epidemic [6], and available literature suggests that most care attrition occurs even before ART initiation occurs, contributing to the World Health Organization’s recent recommendations to offer immediate treatment to all HIV-positive persons worldwide [5, 6, 8–10].

Despite the urgency to improve rapid entry into HIV care and treatment for HIV-positive testers, there are some key gaps in care linkage and retention data from Africa, with most studies focusing on short time periods, single facilities, or retrospective regional data that cannot distinguish the care-seeking patterns of individual patients [11–14]. Falsely low retention rates resulting from patients moving between facilities was highlighted by a Ugandan study, which demonstrated improvement in estimated on-ART retention rates by 19 % after accounting for patients who were receiving care at sites other than their initial site of care linkage [15].

Delayed care entry has previously been associated with higher CD4-positive T-lymphocyte cell count (CD4 count) [16]; sense of well-being, stigma, and transport challenges [7]; male gender, and lack of family disclosure [14, 17]. Care navigators who assist in escorting patients through the care system have been associated with improved linkage and retention [17, 18]. Mental illnesses such as depression and post-traumatic stress disorder (PTSD) have been increasingly demonstrated as factors associated with lower levels of ART adherence in sub-Saharan Africa [19], but have rarely been explored in the context of care linkage [20]. The experience of traumatic lifetime events has also been associated with poor retention in care in the U.S., while at the same time being associated with earlier presentation to care [21].

Herein, we present a prospective study of participants recruited from four different HIV testing centres in northern Tanzania who were followed for 3.5 years with assessments of psychosocial support, depressive and PTSD symptomatology, and documentation of traumatic lifetime events. Our objective was to observe the trajectory of care linkage over the course of the study and to determine associations with care linkage that might ultimately lead to policies to improve engagement after an HIV-positive test. We hypothesized that depressive symptoms, traumatic events, site of testing, and sociodemographic characteristics such as income, age, and gender, would be associated with care linkage. This study is unique in its length of follow up, its recruitment of participants from varied testing sites, and its use of various measures of psychosocial and emotional well-being to assess associations with care linkage and retention, and therefore represents an important contribution to the HIV care linkage literature.

## Methods

### Overview of study design

Coping with HIV/AIDS in Tanzania (CHAT) is a longitudinal cohort study which took place between October, 2008 and April, 2013 with the primary aim of identifying psychosocial factors (including potentially traumatic experiences, depression, social support and sociodemographic factors) associated with adherence to HIV therapy in a resource-limited setting after the roll-out of antiretroviral therapy (ART). A total of 1188 participants were enrolled through August of 2009, 263 of them after having tested HIV-positive at participating HIV voluntary counselling and testing (VCT) centres; these participants (the VCT-positive cohort) are the focus of the analyses in this manuscript. The full study sample size was determined in order to discern differences between participants who were and were not adherent to antiretroviral therapy [22]; the VCT cohort enrolled until the expected number of 750 HIV-positive and a comparator group of 200 HIV-negative individuals had been enrolled into the study. STROBE guidelines were adhered to in the planning and reporting of this research [23].

### Study setting

Participants in the VCT-positive cohort were enrolled from four HIV counselling and testing centres in Moshi (population 184,292), the capital of the Kilimanjaro Region in Northern Tanzania (population 1.6 million; estimated HIV seroprevalence 3.8 %) [24, 25]. Three of the four testing centres were community-based VCT facilities which offered walk-in HIV testing but no HIV treatment services. The fourth testing centre was the Kilimanjaro regional public hospital, which provides walk-in HIV testing as well as provider initiated testing and counselling for patients presenting to the hospital’s outpatient department. The largest regional HIV Care and Treatment Centre (CTC) is also located in the same hospital complex. All persons who test HIV-positive in Tanzania are provided with paper referrals to attend the CTC of their choice. Patients who test positive at the regional hospital and plan to establish care there may be escorted to the on-site CTC if they wish. Throughout the time of this study, there was a rapid expansion in the number of CTCs in Kilimanjaro, ranging from rural health centres to an urban private hospital [26].

### Participant selection and enrolment criteria

Clients aged 18–65 years who resided in three contiguous districts of the Kilimanjaro Region and who tested HIV-positive according to the standard Tanzanian HIV testing algorithm were approached consecutively by trained study staff and offered enrolment into the study. Testers who planned to move outside the study area were excluded.

### Study instrument and follow up

The study was strictly observational and involved biannual interviews by trained study staff. Participants who had not presented to scheduled study visits were traced by phone and in their homes or other contact locations they had provided to the study staff. A targeted interview surrounding date of death, symptoms prior to death, and care-seeking at the end of life was conducted with family members of participants who were reported to have died. Data were entered onto secure netbook computers using Entryware software. Surveys took place at the regional hospital, at a nearby zonal referral hospital, at a local non-governmental organization office in Moshi, or at the participants’ homes. Demographic, family, and relationship characteristics were assessed at each round. Possession of one of the three items associated with the highest quartile of weekly expenditures (a flush toilet, indoor piped water, or a television) was utilized as a measure of wealth for the purposes of this analysis.

At each visit, participants were asked whether they had attended a CTC for their HIV-related care, which CTC they attended, and about ART prescription and adherence. Participants who reported that they had been prescribed antiretroviral therapy were asked to select the medications they had been prescribed using a picture and word chart with commonly prescribed medications. Those in VCT-positive cohort who enrolled into care at the regional hospital or at the northern zonal hospital were also followed by study staff with a biannual clinical survey onto which data from the patients’ hospital charts were extracted. For the last two rounds of surveys, all participants were requested to bring their CTC cards to the study visit. These cards are given to all HIV-positive patients in Tanzania and visit dates, medication prescriptions, World Health Organization HIV clinical stage, and CD4 count results are recorded on them.

Overall performance and symptomatology was assessed using the Physical Composite Score (PCS), calculated from the short form (SF)-8, which has been validated in Africa [27, 28]; a longer form has been utilized for assessment of HIV-related symptomatology in other Africa cohorts [29, 30]. Traumatic life events were measured using the Life Events Checklist, the Childhood Trauma Questionnaire, and other items which assessed the experience of adult and childhood physical and sexual abuse, childhood neglect, experience of conflict or substance abuse in the childhood home, loss of loved ones, and other traumatic events; this has been previously described in detail [31]. In order to assess for depressive severity, the Personal Health Questionnaire (PHQ)-9, which has been widely validated including in sub-Saharan Africa, was administered [32–35]. Post-traumatic stress disorder was ascertained using the PTSD Symptoms Checklist (PCL) [36]. Stigma was measured in two domains utilizing a scale validated in South Africa: attributed stigma, which measured community-level norms; and internalized stigma, which measured HIV-positive persons’ self-stigmatizing beliefs [37]. Social support was measured using a 76-point scale [38].

### Study endpoints

The main goals of this analysis were to describe the overall flow of participants as they engaged in care the 3.5 years of the study and to determine factors associated with failure to link to care within 6 months of an HIV positive test. Participants who delayed entering HIV care for more than 6 months after their diagnosis and those who never entered care throughout the entire 3.5 years of the study were analysed together as one group (delayed/failed care linkage). Care linkage was defined as verbal report of clinic attendance at a recognized Care and Treatment Centre (CTC), but efforts were made to back up the report with concrete data including National HIV Program Identification cards and clinical records. It was noted that several participants died before care linkage data became available and/or presented to acute care for a life-threatening illness rather than presenting for routine HIV care. Therefore, patients who died early were analysed as a separate group. We defined early death as any participant who died prior to their second follow up interview as half of all deaths throughout the 3.5 year follow up occurred during this time frame. Participants who were lost to follow up with no further contact after the baseline study interview were included within the denominator but were not included in the groups of individuals who were deceased or delayed linkage to care. This was because it was impossible to determine whether such individuals had died, never linked to care, or linked to care but did not return for study visits. Determination of ART eligibility was made utilizing available patient data from clinical case report forms and CTC cards, and was based on the Tanzanian National Guidelines [39]. Prior to 2009, the guidelines recommended initiation of antiretroviral therapy for patients with a CD4 count <200 cells/μL or WHO Stage IV clinical illness. These guidelines were revised in January 2009 to additionally recommend ART initiation at <350 cells/μL if a WHO Stage III illness was present. Participants with missing eligibility data who started ART were considered to have been eligible; patients missing eligibility who did not start ART were excluded from the denominator of eligible patients and were recorded separately.

### Data analysis

All analyses were conducted using Stata version 11.0 (Statacorp, College Station, TX, USA). Categorical variables were first analysed using chi-square tests and maximum-likelihood odds ratios. Continuous variables were noted to have skewed distributions and therefore were assessed by comparison of medians utilizing Wilcoxon rank sum test. Because of the relatively small dataset and bias inherent in stepwise models, a predetermined number of factors (approximately 1 per 10 endpoints) thought based on available local and epidemiologic data to be relevant to care linkage were selected for use in a multivariate model. A biprobit multivariable regression model was constructed to explore factors associated with early death and delayed/failed care linkage simultaneously within a joint model. Use of a joint model allowed for determination of possible overlap between the characteristics of the patients who died early and those who delayed in linking to care. Separate logistic models for each dependent variable were created for comparison with the biprobit model.

## Results

**Fig. 1:**
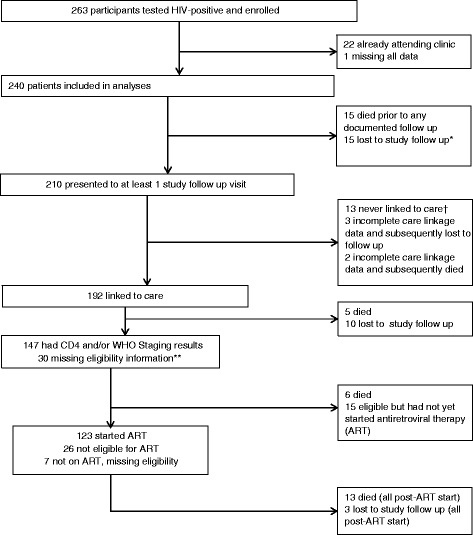
Flow of participants enrolled from four HIV testing centres in Tanzania through 3.5 years of follow up. *Lost to study follow up is defined as participants who no longer attended any study visits or missed their last 2 scheduled study visits. †Participants who followed at study visits but reported never attending an HIV care and treatment center (CTC). **Eligibility for antiretroviral therapy (ART) determined according to local guidelines at the time patients were being followed, described in methods

A total of 263 participants were enrolled between November, 2008 and August, 2009 from four HIV testing centres. Twenty-two patients had tested in order to re-confirm a previously positive test, but were already in care (on ART and/or clinic visit in the last 12 months). These participants were excluded from analyses, as was one additional participant who was missing all data, leaving 240 participants in the final dataset (Fig. 1). Baseline sociodemographic characteristics are displayed in Table 1. Most participants were women (163 (67.9 %) of 240) and had no education or primary education (209, 87.1 %). About half resided in rural areas (123, 51.3 %) and 102 (42.5 %) were married or cohabitating. Linkage to care data was validated by at least one concrete source (CTC card or chart review) for 160 (83.3 %) of the 192 who reported attending clinic.

**Table 1:** Baseline characteristics sociodemographic and testing characteristics of 240 participants enrolled from HIV counseling and testing centers, and univariate associations with early death^a^ or late or no presentation to care^b^

	Total		Early death					Late or no presentation to care				
	N or median	% or IQR^c^	N or median	% or IQR	OR^c^	95 % CI	*p*	N or median	% or IQR	OR	95 % CI	*p*
Total	240		26	10.8 %				40	16.7 %			
Gender												
Female	163	67.9 %	11	6.7 %	1.00			27	16.6 %	1.00		
Male	77	32.1 %	15	19.5 %	3.34	(1.42, 7.81)	0.003	13	16.9 %	0.98	(0.47, 2.02)	0.951
Age, years	36.5	(31, 43)	37.5	(31, 42)			0.772	32	(27, 37.5)			<0.001
Enrollment site												
Hospital OPD^c, d^	149	62.1 %	18	12.1 %	1.00			10	6.7 %	1.00		
Community VCT^c^	91	37.9 %	8	8.8 %	0.70	(0.30, 1.69)	0.701	30	33.0 %	6.84	(2.99, 15.64)	<0.001
Tested due to feeling sick												
No	150	62.5 %	10	6.7 %	1.00			32	21.3 %	1.00		
Yes	89	37.1 %	16	18.0 %	3.07	(1.31, 7.21)	0.007	8	9.0 %	0.36	(0.16, 0.84)	0.014
Marital status												
Married or cohabitating	102	42.5 %	12	11.8 %	1.00			16	15.7 %	1.00		
Widowed	35	14.6 %	4	11.4 %	0.97	(0.29, 3.23)	0.603	5	14.3 %	0.89	(0.30, 2.67)	0.895
Separated or divorced	60	25.0 %	7	11.7 %	0.99	(0.37, 2.68)	0.770	9	15.0 %	0.95	(0.39, 2.31)	0.907
Never married	43	17.9 %	3	7.0 %	0.56	(0.15, 2.12)	0.389	10	23.3 %	1.63	(0.76, 3.98)	0.280
Education												
None or primary	209	87.1 %	22	10.5 %	1.00			30	14.4 %	1.00		
Secondary or higher	31	12.9 %	4	12.9 %	1.25	(0.40, 3.94)	0.692	10	32.3 %	2.84	(1.20, 6.71)	0.013
Primary residence												
Rural	123	51.3 %	13	10.6 %	1.00	1		21	17.1 %	1.00		
Urban	114	47.5 %	10	8.8 %	0.81	(0.34, 1.94)	0.641	19	16.7 %	0.97	(0.49, 1.92)	0.934
Primary occupation												
Business	76	31.7 %	7	9.2 %	1.00			13	17.1 %	1.00		
Farming	39	16.3 %	5	12.8 %	1.45	(0.42, 4.94)	0.551	7	17.9 %	1.06	(0.38, 2.93)	0.910
Non-professional worker (e.g., cleaning)	87	36.3 %	5	5.7 %	0.60	(0.18, 1.99)	0.400	12	13.8 %	0.77	(0.33, 1.83)	0.559
Professional (e.g., nurse)	22	9.2 %	4	18.2 %	2.19	(0.56, 8.44)	0.243	4	18.2 %	1.42	(0.44, 4.59)	0.551
Unemployed	16	6.7 %	5	31.3 %	4.48	(1.15, 17.49)	0.018	4	25.0 %	1.62	(0.44, 5.87)	0.462
3 item wealth asset scale												
0 items	119	49.6 %	10	8.4 %	1.00			17	14.3 %	1.00		
1–3 items	121	50.4 %	16	13.2 %	1.66	(0.72, 3.84)	0.231	23	19.0 %	1.40	(0.71, 2.80)	0.327

^a^Death prior to 2nd study follow up visit

^b^Presented to clinic >6 months after HIV+ diagnosis, or no presentation to clinic

^c^ *Abbreviations*: *OR* odds ratio, *IQR* intraquartile range, *OPD* outpatient department, *VCT* voluntary counseling and testing

^d^Includes both clients who presented specifically requesting an HIV test, and clients tested as part of provider initiated testing and counseling during an outpatient visit

The majority of participants (149, 62.1 %) were enrolled from the regional hospital outpatient department HIV testing centre, with the remaining (91, 37.9 %) enrolled from the three community VCT sites. By 6 months after enrolment, 169 (70.4 %) of 240 participants reported presenting to an HIV care and treatment facility; 41 (17.1 %) delayed more than 6 months, 15 (6.3 %) died, and 15 (6.3 %) were lost to follow up with no further contact after the baseline interview. Twenty-six patients died before their second follow up visit and were analysed in the early death group (10.8 %). Forty-one (17.1 %) participants had not yet sought care at a CTC within 6 months of their enrolment into the study; 27 (11.3 %) eventually linked to care more than 6 months after enrolment, and 13 (5.5 %) remained in the study but did not present to care through 3.5 years of follow up. Among those with available responses, feeling well was the most commonly reported reason for delayed care entry (22 (67 %) of 33), followed by fear of stigma/rejection by a partner (5 (15 %) of 33) or others in the family or community (4 (12 %)).

Bivariable associations between care entry and sociodemographics/testing characteristics (Table 1) and psychosocial measures (Table 2) are displayed. Early death was significantly associated with male gender (OR 3.34; 95 % CI 1.42, 7.81), being unemployed (OR 4.48; 95 % CI 1.15, 17.49), testing due to illness (OR 3.07; 95 % CI 1.31, 7.21), and lower physical performance scores (median 39.3 vs. 42.5, *p* = 0.032). In addition, early death was associated with significantly higher depressive symptomatology (median score 10.7 vs. 9.0, *p* = 0.012) but lower numbers of traumatic events during adult life (median 0 vs. 1 event, *p* = 0.008). On multivariate analysis, male gender (OR 1.72; 95 % CI 1.08, 2.75), testing due to illness (OR 1.63; 95 % CI 1.01, 2.63) and higher depressive symptomatology (a 4 % increased risk of early death for each point of increase in PHQ-9 score; 95 % CI 1–8 %) were associated with early death.

**Table 2:** Associations between psychosocial factors and early death^a^ or late or no presentation to care^b^ among 240 participants enrolled from HIV counselling and testing centres

	Total sample		Early death			Late or no presentation to care		
	Mean	Median	Mean	Median	p*	Mean	Median	p*
SF-8 physical (0–100)^c^	44.2	46.3	39.3	42.5	0.032	44.7	46.4	0.579
PHQ-9 (0–27)^c^	7.6	7.0	10.7	9.0	0.012	7.6	7.0	0.732
PTSD (0–68)^c^	11.1	9.0	9.0	6.5	0.103	11.5	7.0	0.325
Stigma, attributed (0–12)	6.2	7.0	6.0	5.5	0.889	6.5	7.0	0.651
Stigma, internalized (0–44)	18.3	16.0	17.0	17.6	0.879	16.6	16.0	0.803
Support (0–76)	43.7	43.0	47.4	49.5	0.285	43.2	44.0	0.833
Childhood trauma index (0–13)^d^	1.8	2.0	1.5	1.0	0.226	1.8	2.0	0.014
Adult trauma index (0–5)^d^	0.79	1.0	0.4	0.0	0.008	0.9	1.0	0.341

^*^Compares results of psychosocial/stigma scales for participants with early death to the total sample, and participants with late presentation to care to the total sample. Those with early death were not compared directly to those with late/no care presentation

^a^Death prior to 2nd study follow up visit

^b^Presented to clinic >6 months after HIV+ diagnosis, or no presentation to clinic

^c^ *Abbreviations*: *SF8* 8 question short from of the of physical performance scale--see manuscript text for details, higher scores indicate higher functioning, *PHQ-9* personal health quesionnaire-9, assesses depression, higher scores indicate higher depression levels, *PTSD* post-traumatic stress disorder, higher scores indicate higher levels of PTSD symptoms

^d^Childhood and adult trauma index correspond to the number of study-assessed traumatic events that occurred in childhood or adulthood

Delayed care linkage or never presenting to care was significantly associated with testing at a community facility (OR 6.84; 95 % CI 2.99, 15.64), younger age (median 32 vs. 36.5 years, *p* <0.001), secondary education (OR 2.84, 95 % CI 1.20, 6.71), lower childhood trauma scores (*p* = 0.014), and illness at the time of testing (negative association, OR 0.36, 95 % CI 0.16, 0.84) (Tables 1 and 2). On multivariate analysis (Table 3), community testing site remained significantly associated with delayed care linkage (OR 2.89; 95 % CI 1.79, 4.66). Testing due to illness was also negatively associated with delayed care linkage (OR 0.58; 95 % CI 0.34, 0.96). Age retained borderline significance (2 % decrease in risk of delayed or failed care linkage for each year increase in age; 95 % CI 0–5 %).

**Table 3:** Multivariate analysis of associations with early death^a^ or late or no presentation to care^b^ among 237^c^ participants enrolled from HIV counselling and testing centres

	Early death			Late or no presentation to care		
	OR	95 % CI	*p*	OR	95 % CI	*p*
Male gender	1.72	(1.08, 2.75)	0.022	1.15	(0.73, 1.83)	0.540
Age	0.99	(0.96, 1.02)	0.566	0.98	(0.95, 1.00)	0.057
Community enrollment site	0.76	(0.44, 1.30)	0.257	2.89	(1.79, 4.66)	<0.0001
Tested due to illness	1.63	(1.01, 2.63)	0.044	0.58	(0.34, 0.96)	0.034
PHQ-9	1.04	(1.01, 1.08)	0.012	0.99	(0.96, 1.03)	0.680
Stigma, internalized	1.00	(0.93, 1.04)	0.551	0.98	(0.93, 1.03)	0.368

*Abbreviations*: *OR* odds ratio, *CI* confidence interval, *PHQ-9* personal health quesionnaire-9 (assesses depression, higher scores indicate higher depression levels)

^a^death prior to 2nd study follow up visit

^b^presented to clinic >6 months after HIV+ diagnosis, or no presentation to clinic

^c^participants with missing data in any measure were excluded

**Fig. 2:**
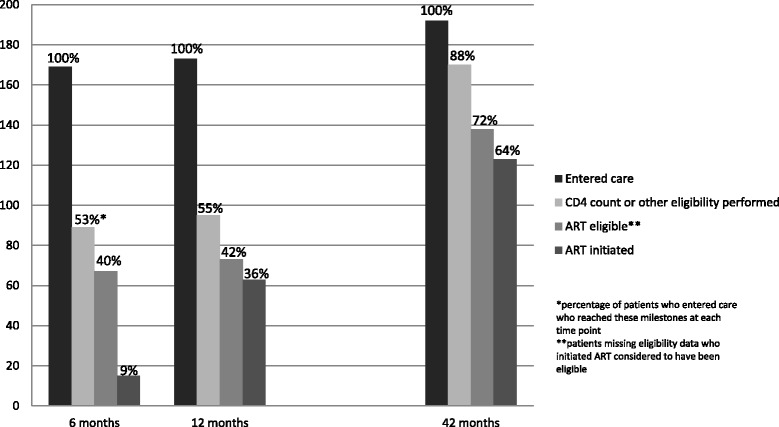
Cascade of care entry, CD4 testing and antiretroviral therapy (ART) initiation at 6, 12, and 42 months among 240 patients who tested HIV-positive

A total of 169 patients (70.4 %) were retained in the cohort for at least one follow up visit and reported linkage to care within six months of their initial HIV-positive test. Of these, 89 (57.1 %) participants had documented CD4 cell count or other eligibility criteria performed within 6 months of their initial positive test and 67 (75.3 %) of those with CD4 cell count results were eligible for ART (Fig. 2). However only 15 (22.3 %) of those eligible by CD4 initiated ART within 6 months of diagnosis. By 12 months after enrolment, just 4 additional participants had linked to care, but the number who had started ART increased to 63 (86.3 % of those documented to be eligible). By the end of the 3.5 year study, 192 participants had presented to care, and 123 (89.1 % of those eligible and 64.0 % of the linked cohort) had started ART. Among patients who reported linkage to care but were never prescribed ART (*n* = 69), 26 (38 %) were not eligible, 15 (22 %) were eligible but had not initiated ART, 11 (16 %) died prior to ART initiation, 10 (14 %) were lost to study follow up prior to initiation of ART, and 7 (10 %) were missing eligibility information. Participants who tested HIV-positive at community testing sites were significantly less likely to have ART eligibility data available (OR 0.52; 95 % CI 0.30, 0.90) and less likely to report initiation of ART at any time during the study (OR 0.58; 95 % CI 0.34, 0.98) compared to those who tested HIV-positive at the regional hospital outpatient department.

## Discussion

In this prospective study of care linkage and retention among a cohort of 240 participants enrolled from distinct HIV counselling and testing sites in northern Tanzania, 30 % of patients were lost to study follow up or not in care within six months of their HIV diagnosis, which is similar to data previously described in sub-Saharan Africa in cross-sectional or shorter term prospective studies [5, 6]. Importantly, the prime factor associated with care linkage in our study in both bivariable and multivariate analyses was testing site, with patients who tested HIV-positive at community-based facilities more than twice as likely to delay care linkage more than 6 months or to never link to care throughout 3.5 years of study follow up. Similarly, lower levels of ART assessment and ART initiation were noted in the group of participants who enrolled from community sites compared to those who enrolled at the regional hospital outpatient department. This association between community-based testing and delayed/failed care linkage held even after adjusting for factors such as age, gender and illness which might have distinguished those who opted to test in the community from those who tested at the hospital outpatient department. To our knowledge, this has not been previously documented in the literature as part of a prospective cohort study, and it complements other studies in diverse environments which demonstrate that co-location of testing and care services, and/or direct facilitation (care navigation) improve care linkage and ART initiation [40–42]. Despite the fact that the community testing sites in Moshi from which our study participants were recruited were within a 2 km radius of the regional hospital CTC, escorting the participant to CTC, as is often done for hospital outpatient department testers, appears to have additional value. It is possible that people who test in healthcare environments feel more comfortable accessing care, or that there are other unmeasured differences between those who decide to test at community-based versus health care based centres, however the strength of our association after adjusting for other factors suggests that it is a key independent factor in care linkage. It is also noteworthy that the great majority of patients who linked to care throughout the study did so within 6 months of their positive test; therefore, that early period is critical in establishing care practices over the coming years.

As is the case with many other care linkage studies in Africa, our data reveal gaps not just in patient presentation to care, but in assessment of ART eligibility and initiation of ART [12–14]. This was particularly the case early on in the study, with only about 10 % of participants who linked to care initiating ART within 6 months. Nonetheless these proportions increased steadily throughout the study such that nearly 90 % of participants with documented eligibility had initiated ART by the end of the study. Potential adoption of new international guidelines to initiate all patients on ART regardless of CD4 cell count may have major benefits in reducing such delays.

As evidenced by the early mortality in this and all linkage to care studies in sub-Saharan Africa, immediate care entry and ART initiation may have lifesaving potential. Additionally, a consistent community and healthcare policy message that all people with HIV should start ART might assist in curbing the dangerous belief that there is no need to present to testing and/or care until one is ill. This belief was pervasive among our study participants who delayed care, and may also have been present among those who died within six months of enrolment, significantly more of whom tested due to illness. A recent qualitative study identified many of these concerns among Kenyans living with HIV; one theme that emerged was patients’ belief that ART was something only necessary during dire illness [43].

Increased severity of depressive symptoms was significantly and independently associated with early death, but not with late linkage to care. Early death may have been a surrogate for testing late in the course of illness, and it is possible that testing late in the course of illness may have been associated with depression. In addition, despite its independent association with early death on multivariate analysis, given the lower physical performance scores and association with testing due to illness in the early death group, depression in this group may have been a result of physical illness.

Surprisingly, most of the other mental health and psychosocial measures explored as predictors of care linkage and retention showed no significant association in this study. Several factors may have contributed to this finding. Baseline interviews sometimes took place on the day of testing and other times up to a few weeks after the positive test. This variation in and of itself could have impacted responses to sensitive items such as stigma, depression and PTSD, thereby biasing effects estimates. In our study, traumatic events were associated with better linkage to care. This is consistent with data from the U.S. which found that childhood trauma increased health-seeking behaviour [21].

The finding that men were more likely to experience early death in this study reflect findings from other sub-Saharan African studies which demonstrate that men are often more difficult to engage for testing [44, 45], more likely to delay care linkage [14, 17], and more likely to drop out of care [46]. Further research is needed to understand the unique concerns of men and to adapt appropriate linkage and retention efforts targeted towards them.

This study has several important strengths, including the long length of follow up, the comprehensive nature of the survey instrument, and the recruitment of participants from several different HIV testing sites. Several weaknesses are also important to note. Linkage to care was documented in patients who followed in study-affiliated clinics or who presented with their CTC card, but was reported only verbally in 17 % of cases and may have been subject to social desirability bias. Nonetheless, very detailed questions were asked about site of care linkage, names of ART medications, etc., helping to validate the responses that were received. The relatively small numbers patients who had died or failed/delayed care linkage limited the power of the study to distinguish the unique characteristics of each of these groups, which may be quite important in further delineating risks for poor follow up and measures to improve it. Information about clients who opted to participate in the study versus those who declined participation is not available; selection bias may have incurred for patients enrolled were actually more likely to follow up and therefore estimates provided may be overestimates of actual levels of follow up. Finally, given the similarity of themes identified in this study compared to other sub-Saharan African settings, we believe that these results are generalizable to other sub-Saharan African settings with similar set-ups (HIV testing offered in various different locales without mandatory patient navigation).

## Conclusions

Our prospective 3.5 year study demonstrated that testing site strongly impacted linkage to care in Tanzania, with testers at community based sites more likely to delay or fail to present for care than those who tested at the hospital outpatient clinic. Targeting linkage efforts towards community-based centres and co-locating HIV testing and care should be an urgent public health priority. In addition, marked delays in initiation of ART were evident even among patients who linked to care quickly after diagnosis. We hope that our results strengthen movements to co-locate testing and treatment, to offer navigation services, and to expand immediate provision of ART.

## Abbreviations

ART: Antiretroviral therapy
CHAT: Coping with HIV/AIDS in Tanzania
CI: Confidence interval
CTC: Care and Treatment Centre
HIV: Human Immunodeficiency Virus
OR: Odds ratio
PCS: Physical composite score
PTSD: Post-traumatic stress disorder
USD: United States dollars
VCT: Voluntary counselling and testing
WHO: World Health Organization
